# Idiotism and Aphonia, from Fright during Pregnancy

**Published:** 1830-10-01

**Authors:** 


### XXXV.

### IDIOTISM AND APHONIA, FROM FRIGHT, DURING UTERO-GESTATION.

THE following curious case, related by Madame Boivin, the famous Parisian midwife, had escaped our notice in the Journal Complementaire, and we take it from the American Journal of the Medical Sciences for May, of this year.

"A female, aged twenty-three, nervous constitution, bilious temperament, dark brunette, a mother at the age of twenty, her labour taking place at the seventh month of pregnancy, in consequence of bad treatment from her parents. The accouchement was preceded and followed by an abundant metrorrhagia and frequent faintings. Her health was however restored, and she left her paternal mansion and went to Paris. She again became pregnant, and at the sixth month of utero-gestation she became extremely jealous, violently reproached and menaced her lover, who after vain attempts to calm her, threatened to have her removed from his dwelling by force, and went out to seek a police officer. The patient immediately hid herself, and it was not until after a long search that she was afterwards found in a closet of a wardrobe. She was found in a state of stupor, which was at first supposed to be feigned. But nothing that could be said could induce her to leave her retreat or change her attitude or expression. It was necessary to remove her, and a physician was called, who thought that the patient was feigning. She however did not speak, nor make any signs; she did not exhibit any wants, and preserved any position in which she was placed. In this state she was taken to *La Maison Royal de Santé*, August 20th, 1820, two days after the occurrence, and was placed under the care of M. Duméril. She was here carefully watched by a special attendant. Her aspect at this period was frightful—her black hair stood erect on her head—her dark face, thin and excessively pale—her large black eyes, surmounted by thick eyebrows of the same colour, were fixed and prominent—her mouth wide open, and her chin resting on her chest. When any one entered her chamber, she turned her head, looked aside with an expression of fear, afterwards examined the person attentively as long as they continued in the room. She did not speak nor move; it appeared as if she did not understand any thing that was said to her. Her appearance was so hideous that she was called by the other patients the vampire.

This singular state was unaccompanied with fever. An infusion of orange leaves, and a draught with ether, were ordered; but twenty-four hours elapsed without its being possible to induce her to take a cup of this liquid: she had not taken any thing during the preceding two days. In the course of the fourth day, the sixth from the attack, she refused as usual the drinks which were offered her; but she made signs that she wanted an empty bottle that was in her sight, which was given her, when she put into it some of the fluid, and drank for the first time during six days. She had only urinated once, and then in her bed, and had not slept during this period. She continued afterwards to take drinks, but only with the bottle. A *douche ascendante* produced an evacuation from her bowels for the first time in six days.

The fifth day she took a little soup, and she was taken into the garden. She sat upon the ground, collected together the sand, selected the pebbles and shells that she found in it, and amused herself as if she were an infant of two years of age. Some one pretended to wish to take them away from her; her face immediately took on an expression of great anger, and afterwards she wept heartily. When the pebbles were returned to her, she exhibited a stupid joy. She continued this puerile amusement during twelve days; her speech did not return, and she did not give any sign of a return to reason.

The sixth day a large blister was applied to her neck, but neither during the drawing or dressing did she exhibit any signs of pain.

On the eighth, the shower bath was ordered. The patient on being stripped entirely of her clothes, made no resistance. When the water fell upon her she appeared seized with fright; her limbs were violently agitated; but she did not attempt either to raise or remove herself; she neither groaned nor cried; her respiration became suspended; finally, syncope came on, from which she was with difficulty revived. After being placed in bed, she slept many hours. She showed afterwards by signs that she remembered the shower bath.

On the twelfth day she showed by signs that she had pains in her right leg. A flying blister was applied to her knee, and the pain disappeared. From this period she attempted to speak; she moved her lips, but she did not articulate a single word.

The fifteenth day, when she was asked how she had passed the preceding night, she took a piece of paper and pencil out of the hands of the interrogator, and wrote down an answer. She asked at the same time where she was; and appeared much astonished on being told that she was in a hospital in Paris. During three days she communicated in this way with her attendants.

On the nineteenth day she was attacked with pain in her back, which becoming more violent, she uttered a shriek and afterwards exclaimed, 'Ah! mon Dieu!' The attendants surprised and frightened, repeated the exclamation, and ran into the passages and wards, crying the vampire has spoken. The patient in her turn appeared equally surprised at what was passing around her; her attendants on recovering from their surprise, re-entered her chamber to congratulate her. She inquired of them what there was extraordinary about her? whether she had not spoken before, and said she believed that they had been deaf. When her history and appearance were detailed to her she joined heartily in the laugh.

From this period she continued to speak with facility, and even very agreeably. Her appearance became very pleasant, and her eyes assumed an expression of the utmost mildness. She did not recollect any of the circumstances which had preceded or attended the disease; not even the shower bath, which had produced so violent an effect.

The sight of her lover, who visited her during her disease, did not produce any effect upon the patient; she regarded him at first with fright, as she did others.

The patient remained in the hospital until after her accouchement, when she was discharged well."

---

